# Robust Remote Sensing of Trace‐Level Heavy‐Metal Contaminants in Water Using Laser Filaments

**DOI:** 10.1002/gch2.201800070

**Published:** 2018-10-29

**Authors:** Helong Li, Hongwei Zang, Huailiang Xu, Hong‐Bo Sun, Andrius Baltuška, Pavel Polynkin

**Affiliations:** ^1^ State Key Laboratory of Integrated Optoelectronics College of Electronic Science and Engineering Jilin University 2699 Qianjin Street Changchun 130012 China; ^2^ State Key Lab of Precision Measurement and Instruments Department of Precision Instrument Tsinghua University Haidian Beijing 100084 China; ^3^ Photonics Institute Vienna University of Technology A‐1040 Vienna Austria; ^4^ College of Optical Sciences The University of Arizona Tucson AZ 85721 USA

**Keywords:** femtosecond laser pulse, filamentation, heavy‐metal pollution, remote sensing

## Abstract

Water is the major natural resource that enables life on our planet. Rapid detection of water pollution that occurs due to both human activity and natural cataclysms is imperative for environmental protection. Analytical chemistry–based techniques are generally not suitable for rapid monitoring because they involve collection of water samples and analysis in a laboratory. Laser‐based approaches such as laser‐induced breakdown spectroscopy (LIBS) may offer a powerful alternative, yet conventional LIBS relies on the use of tightly focused laser beams, requiring a stable air–water interface in a controlled environment. Reported here is a proof‐of‐principle, quantitative, simultaneous measurement of several representative heavy‐metal contaminants in water, at ppm‐level concentrations, using ultraintense femtosecond laser pulses propagating in air in the filamentation regime. This approach is straightforwardly extendable to kilometer‐scale standoff distances, under adverse atmospheric conditions and is insensitive to the movements of the water surface due to the topography and water waves.

Pollution of natural bodies of water poses a fundamental threat to all forms of life on this planet. A specific form of pollution is by heavy‐metal elements such as lead, copper, cadmium, mercury, and arsenic, the use of which is widespread in large‐scale industries including mining and fertilizer manufacturing. Industrial accidents leading to spills of toxic heavy‐metal contaminants continue to happen; therefore, the detection of such contaminants is critical for environmental protection. An ideal detection approach needs to be: (i) sensitive to parts‐per‐million‐level (ppm) or lower concentrations; (ii) rapid, as water pollutants are mobile and spread rapidly after their introduction at a particular location, through diffusion and water flow; (iii) capable of simultaneous detection of multiple contaminants; (iv) remote.

No detection technique demonstrated to‐date satisfies all of the abovementioned requirements. The established techniques and procedures based on the methods of analytical chemistry rely on the collection of water samples and their subsequent analysis in a laboratory, which is neither rapid nor remotely implementable.^1^, ^2^, ^3^, ^4^ Laser‐induced breakdown spectroscopy (LIBS) has been previously applied to the detection of water contaminants at the ppm‐level concentrations, which is the sensitivity sufficient for arranging a timely response to industrial spill events.^5^, ^6^, ^7^, ^8^, ^9^, ^10^ The drawback of the conventional LIBS schemes is that they rely on the use of pulsed nanosecond or femtosecond laser beams that are tightly focused on the air–water interface, in order to reach the necessary laser fluence on the order of multiple Joules per cm^2^. Therefore, setups involving tight beam focusing can only be implemented in a controlled laboratory environment with a stable air–water interface, whether nanosecond or femtosecond pulses are used.^11^

Here we report the results of proof‐of‐principle experiments on the application of an alternative laser‐based approach to the simultaneous detection of multiple heavy‐metal contaminants in water, at the ppm‐level concentrations. Instead of relying on tight focusing of the laser beam, we use intense femtosecond optical pulses that propagate in the air in the self‐guiding regime commonly termed laser filamentation.^9^, ^10^, ^12^, ^13^, ^14^, ^15^ The dynamic balance between self‐focusing and plasma defocusing and nonlinear losses in laser filaments results in the generation of meters‐long light strings with sustained optical intensity on the order of 10^14^ W cm^−2^.^13^, ^14^, ^15^ Filaments can be produced at a desired standoff distance that can be in the multikilometer range, and in adverse propagation conditions.^16^, ^17^, ^18^, ^19^, ^20^ Using filaments for remote sensing of solid (100% concentration) copper or aluminum has been reported previously.^21^, ^22^ However, remote LIBS detection of trace amounts of water contaminants, using femtosecond laser filaments, has not been yet reported. Here we combine the advantages of the LIBS scheme for trace element detection of water and the remote generation capability of high laser intensity in long femtosecond light filaments, and show simulataneous measurements of multiple water pollutants, with ppm‐level accuracy. We further demonstrate robustness of this technique against simulated water waves for field measurements.

Our laboratory‐scale demonstration is straightforwardly extendable to the kilometer‐scale standoff distances, thanks to the abovementioned remarkable properties of femtosecond laser filaments. As we will show below, our femtosecond approach is insensitive to the variations of the air–water interface that may occur, under realistic outdoor conditions, due to the topography and water waves. Various heavy‐metal pollutants can be detected rapidly and remotely, e.g., from an airplane, as conceptualized in **Figure** **1**a. Our approach is ideal for prompt detection of emergency toxic spill‐off situations that require immediate response.

**Figure 1:**
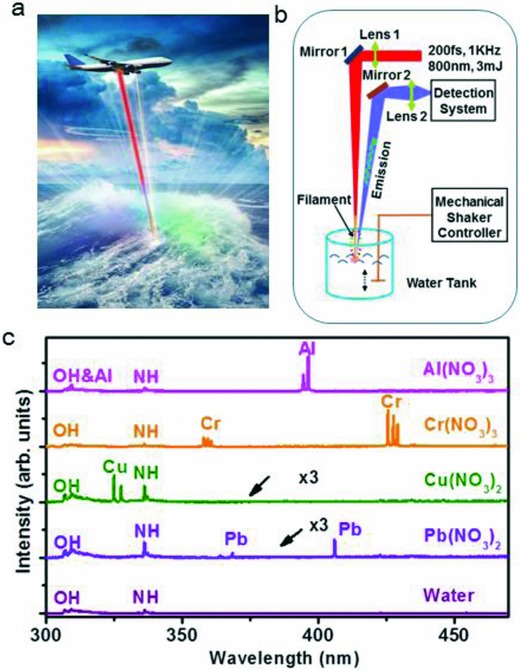
Illustration of the remote sensing concept based on the application of self‐channeling femtosecond laser pulses and its laboratory‐scale demonstration. a) Illustration of the remote detection of water contamination from an airplane. b) Schematic of the experimental setup used in this work (see Experimental Section). c) Detected spectra of five samples of contaminated water. (Individual spectra are shifted vertically, for clarity.)

With the experimental setup shown in Figure 1b (for details, see Experimental Section), in Figure 1c, we show the experimentally recorded spectra of emission resulting from the excitation of the water surface by a laser filament. Different spectra correspond to the samples of pure deionized water contaminated by 500 ppm concentrations of different salts: Al(NO_3_)_3_, Cr(NO_3_)_3_, Cu(NO_3_)_2_, and Pb(NO_3_)_2_. Plasma produced through strong‐field ionization of water and contaminants is cold and short‐lived. It emits clean spectral lines of the chemical species of interest (see Supporting Information).

Different spectral lines shown in Figure 1c are unambiguously assigned to the metal contaminant ions introduced into the water samples, e.g., Al at 396.2 nm (*3s^2^4s*→*3s^2^3p* transition), Cr at 425.4 nm (*3d^5^4p*→*3d^5^4s*), Cu at 324.8 nm (*3d^10^4p*→*3d^10^4s*), and Pb at 405.9 nm (*6s^2^6p7s*→*6s^2^6p^2^*) (see Supporting Information). In addition, evident in the spectra are the emissions by free radicals OH at 307 nm (*A* ^2^ *Σ* ^+^→*X* ^2^ *Π*) and NH at 336 nm (*A* ^3^ *Π*→*X* ^3^ *Σ* ^−^). These compounds are formed through complex photochemical processes involving water and air molecules.^23^, ^24^, ^25^ Referencing the amplitudes of the detected spectral lines to that of the OH line, which corresponds to the known ≈1 000 000 ppm water concentration, can be used for the quantitative calibration of the pollutant concentrations.

In **Figure** **2**, we show experimental data for the intensities of the representative spectral lines of four different heavy‐metal contaminants dissolved in the water samples, as functions of their concentrations. We define the limits of detection (LODs) for these elements, according to the International Union of Pure and Applied Chemistry (IUPAC), as LOD = 3σ_BG_ S^−1^, where σ_BG_ is the standard deviation of the signal and S is the slope of the calibration curve for the signal versus concentration. Based on the data, the LODs for the four heavy‐metal pollutants are found to be 1.69, 1.38, 9.91, and 15.31 ppm for Al, Cr, Cu, and Pb, respectively. These results show that the filament‐based LIBS is capable of trace‐level sensitivity, similar to the sensitivity attainable with conventional fs‐LIBS that involves tight focusing of the laser beam.^9^, ^10^

**Figure 2:**
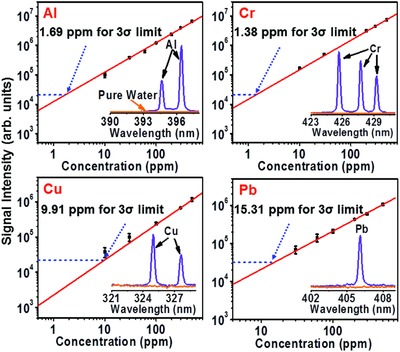
Measurements of ppm‐level metal concentrations. Concentrations of the Al, Cr, Cu, and Pb pollutions in water measured from a target 1.1 m away. The extrapolation of the data for each element yields the corresponding limit of detection (LOD), defined according to the International Union of Pure and Applied Chemistry (IUPAC).

The data shown in Figure 2 are obtained with a single dissolved heavy‐metal pollutant in each water sample. However, our approach is straightforwardly capable of detecting several pollutants simultaneously, as shown in **Figure** **3**. Here, four representative heavy metals, all at 50 ppm concentrations, are detected simultaneously in a common water sample. The spectral lines corresponding to these elements are clearly identifiable in the measured spectrum, with the relative amplitudes of the spectral peaks consistent with the data shown in Figure 2 for the cases when each sample had only a single variety of dissolved pollutant.

**Figure 3:**
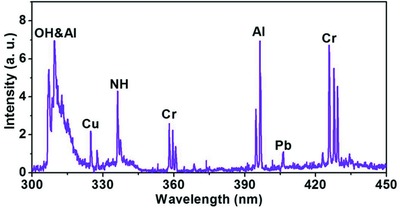
Simultaneous detection of multiple trace metals in water. The detected spectrum of emission from water simultaneously contaminated by Al, Cr, Cu, and Pb, at the concentration of 50 ppm for each element.

As pointed out above, the key advantage of the femtosecond filament–based detection approach is that it is extremely tolerant to the variations of the distance between the laser source and the water surface. This advantage is demonstrated by the data shown in **Figure** **4**. Here, we use a water sample contaminated with 500 ppm concentration of Al(NO_3_)_3_, with the representative spectral line at 396.2 nm, and vary the distance between the focusing lens and the water surface. In these experiments, we use two different lenses. The first lens, with the focal length of 25 cm, produces tight focusing with high intensity confined to the Rayleigh range of the laser beam, which is representative of the conventional LIBS approach. In that case, the signals are highly sensitive to the distance between the focusing optic and the water surface, as follows from the data shown with circles in Figure 4. The second lens has a focal length of 100 cm and generates loose focusing of the laser beam. Beam propagation in this case is dominated by nonlinear self‐channeling. As a result, the detected signal is insensitive to the variation of the distance between the focusing optic and the water surface, as long as that variation does not exceed the length of the laser filament, which is ≈6 cm in this demonstration. The corresponding data are shown with stars in Figure 4.

**Figure 4:**
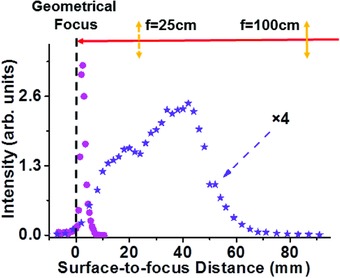
Detected spectra in the case of tight and loose focusing of the laser beam. The amplitude of the Al spectral line versus distance between the focal plane and the water surface, for two different focusing lenses, with focal lengths of 25 cm (circles) and 100 cm (stars).

To further illustrate the utility of our approach under adverse conditions, we conducted our experiment in a water tank with stimulated surface waves. The disturbances of the water surface, with various amplitudes and frequencies, are induced by a water‐immersed mechanical shaker. Videos of the experiment can be found in the Supporting Information. In this case, the water samples are contaminated with Al(NO_3_)_3_ at various concentrations ranging from 10 to 500 ppm. The signals recorded from the undulating water surface, for each concentration, are shown in **Figure** **5**. They are weakly dependent on the parameters of the mechanical excitation imposed by the shaker, demonstrating robustness of this sensing approach against disturbances caused by water waves.

**Figure 5:**
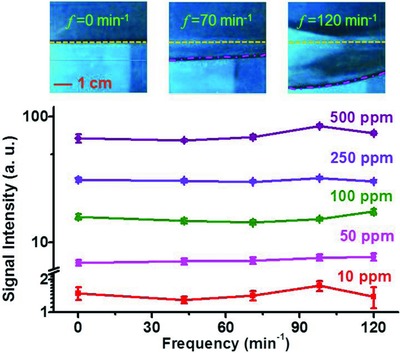
Insensitivity of the filament‐based remote sensing to the disturbances of the water surface. The amplitude of the Al spectral line as a function of the vibration frequency of the mechanical shaker immersed in the water, for Al concentrations of 10, 50, 100, 250, and 500 ppm. The three images at the top of the figure show snapshots of the undulating water surface for the cases of vibration frequencies of 0, 70, and 120 min^−1^. The dot‐dashed and dashed lines show the water surface with the vibration turned on and off, respectively.

In the long‐range implementation of the laser filament–based sensing of water pollutants, the filament will be produced, at a remote water surface, through the self‐focusing collapse of a temporally prechirped and collimated, instead of a weakly focused, laser beam. The filamentation zone, with the sustained clamped laser intensity on the order of 50 TW cm^−2^, will be several meters long, followed by the postfilamentation zone, where intensity is also substantial.^26^, ^27^ The span of the natural filaments is comparable to the amplitude of the natural wind–generated water waves, thus enabling rapid remote detection of the heavy‐metal pollutants in natural bodies of water from a flying airplane (see Figure 1a), from a kilometer‐scale distance.^28^ Due to intensity clamping, the level of the optical signal generated at the point of emission on the water surface will be similar to that in our laboratory‐scale demonstration. Note that although the particular value of clamped intensity in the long‐range laser filament may vary depending on the humidity of air and other environmental conditions, our spectroscopic measurement still can be made quantitative by referencing the spectral peaks to the spectral signature of water.^29^ The level of signal detected from an ≈100 m distance can be made comparable to the one in our laboratory‐scale demonstration using a correspondingly larger signal‐collection optic (see Supporting Information). In addition, adaptive optics and spatiotemporal focusing, among other techniques, could be empolyed to enhance the intensity of the LIBS signal, leading to higher sensitivity.^30^, ^31^

In summary, we have introduced a new approach for the remote detection of trace amounts of multiple heavy metal ion pollutants in water. The key advantages of this technique derive from the properties of laser filaments in air, e. g., the sustained high level of optical intensity inside the longitudinally extended filament core and the relative immunity of filamentation to adverse atmospheric conditions. Through referencing the spectroscopic signatures of the pollutants to that of the water itself, pollutant concentrations can be quantitatively measured with the ppm‐level accuracy. We have conducted a proof‐of‐principle demonstration of our approach on a laboratory scale and discussed the scalability of this technique to the practically relevant standoff distances. Potential applications of this approach are in remote sensing of heavy‐metal contaminants in the natural bodies of water polluted by sewage and industrial waste.

## Experimental Section

In the experiments, 200 femtosecond‐long laser pulses at the center wavelength of 800 nm were generated, at the rate of 1000 pulses per second, by a commercial Ti:Sapphire laser system (Spectra Physics, Spitfire ACE) (see Figure 1b). The energy per laser pulse was 3 mJ. The laser beam was reflected by a 45° mirror (Mirror 1) and propagated vertically downwards. The beam was weakly focused with a 100 cm focal‐length lens (Lens 1), onto the surface of water inside an open water tank. The self‐focusing of the beam in the air resulted in the formation of an ≈6 cm long laser filament. The water tank was positioned so that the filament started ≈3 cm above the water surface and terminated shortly upon entering the water. The water waves were introduced by a water‐immersed mechanical shaker that generated vibrations at the frequency in the range 0–150 per minute. Optical emission from the plasma, produced on the water surface through the interaction with the laser filament, was collected at a distance of 110 cm from the excitation point on the water surface, by an Aluminium mirror (Mirror 2) and a 5.08 cm diameter lens (Lens 2) with a focal length of 6 cm. The collecting optic focused the emission onto the 100 mm wide entrance slit of a spectrometer (Andor Shamrock SR‐303i), which was equipped with a 1200 lines per millimeter grating and a gated, intensified CCD camera (Andor iStar ICCD). The spectral data were 100 000 laser shots (100 s integration time). The standard deviation was determined over the sample of three measurements. In all measurements reported here, the ICCD gate opened 200 ns after the arrival of the direct scattering of the excitation laser pulse to the detection system, and the temporal width of the ICCD gate was set to 1 µs. (See Supporting Information for the discussion of the gating parameter optimization.)

## Conflict of Interest

The authors declare no conflict of interest.

## Supporting information

Supplementary
