# Supplementary material for: Robust Remote Sensing of Trace‐Level Heavy‐Metal Contaminants in Water Using Laser Filaments
Source: Glob Chall. 2018 Oct 29;3(1):1800070. doi: 10.1002/gch2.201800070 (PMC6383966; doi:10.1002/gch2.201800070)
Supplement: Supplementary file 1 — Supplementary [file GCH2-3-1800070-s001.pdf]

Copyright WILEY-VCH Verlag GmbH & Co. KGaA, 69469 Weinheim, Germany, 2018.

Global Challenges
Open Access

Supporting Information

for *Global Challenges,* DOI: 10.1002/gch2.201800070

Robust Remote Sensing of Trace-Level Heavy-Metal Contaminants in Water Using Laser Filaments

Helong Li, Hongwei Zang, Huailiang Xu, Hong-Bo Sun,* Andrius Baltuška, and Pavel Polynkin*

Supporting Information

Robust remote sensing of trace-level heavy-metal contaminants in water using laser filaments

Helong Li, Huailiang Xu, Hongwei Zang, Hong-Bo Sun*, Andrius Baltuška and Pavel Polynkin*

1. Temporal gating of the signal collection

In Figure S1, we show the emission spectra obtained with different ICCD gate delays. The gate delay is measured relative to the moment t=0 when the laser pulse arrives at the target. All spectra are measured with a fixed gate width of 1 microsecond. The Al concentration is 500 ppm. It can be seen from Figure S1 that because plasma excited through the interaction with an ultrashort laser pulse is cold, the useful Al spectral signal can be cleanly separated from the thermal emission background.

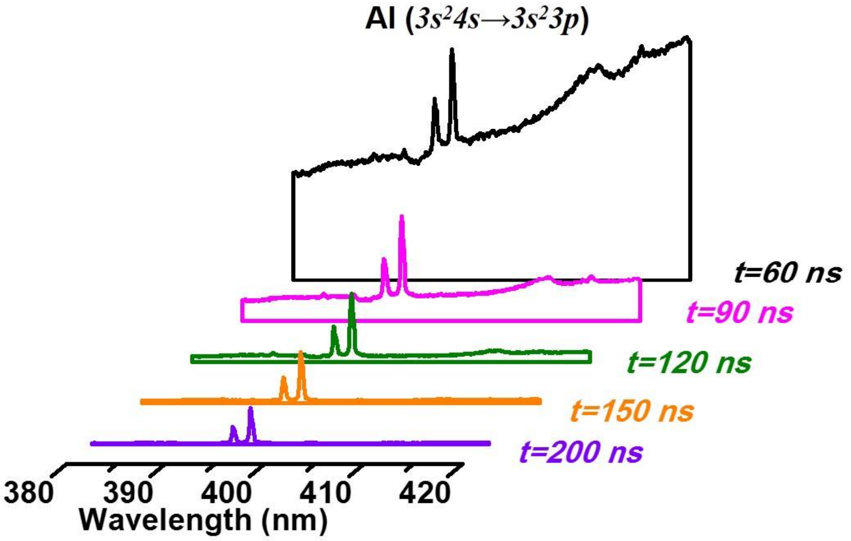

Supplementary Figure S1. Optimization of the gate delay. The ICCD gate width is set to 1 μs, and the gate delay varies from 60 ns to 200 ns. When gate delay is set to 200

ns, thermal background is no longer observed.

In Figure S2, we show the spectral signals for 500 ppm concentrations of four metals in water as a function of the ICCD delay time. The gate width is fixed at 1 μs. Based on these data, we conclude that the gate width of 1 μs is sufficient to collect all useful signals on the spectral lines of interest.

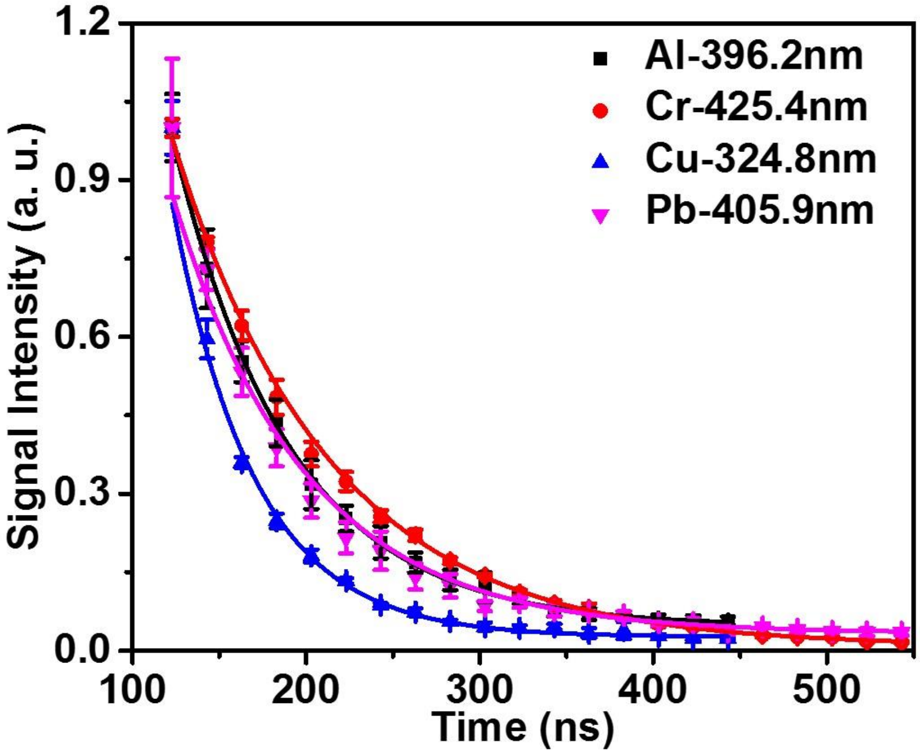

Supplementary Figure S2. Detected signal with a fixed ICCD gate width of 1 μs, vs. ICCD delay time. After the delay of 200 ns, the thermal emission component in the signal is negligible, according to Figure S1. Subsequent useful signal on the spectral lines of interest can be completely collected within the chosen gate width.

2. Assignment of the observed spectral lines

The spectral lines shown in Fig. 1 of the manuscript are summarized in Table S1. The lines are assigned to the transitions in Al I, Cr I, Cu I, and Pb I according to the NIST database of atomic spectra (https://www.nist.gov/pml/atomic-spectra-database), as shown in Table S1.

Table S1. Observed wavelength and the corresponding levels and transitions

	Upper Level			Lower Level			Calculated Wavelength in air	Observed Wavelength in air
	Conf.	J	E(cm^{-1})	Conf.	J	E(cm^{-1})	λ(nm)	λ(nm)
**Al**	$3s^23d$	5/2	32436.80	$3s^23p$	3/2	112.06	309.27	309.3
	$3s^24s$	1/2	25347.76	$3s^23p$	1/2	0.00	394.40	394.4
	$3s^24s$	1/2	25347.76	$3s^23p$	3/2	112.06	396.15	396.2
**Cr**	$3d^44s4p$	4	27935.24	$3d^54s$	3	0.00	357.87	357.8
	$3d^44s4p$	3	27820.20	$3d^54s$	3	0.00	359.35	359.3
	$3d^44s4p$	2	27728.81	$3d^54s$	3	0.00	360.53	360.5
	$3d^54p$	4	23498.82	$3d^54s$	3	0.00	425.44	425.4
	$3d^54p$	3	23386.34	$3d^54s$	3	0.00	427.48	427.5
	$3d^54p$	2	23305.00	$3d^54s$	3	0.00	428.97	429.0
**Cu**	$3d^{10}4p$	3/2	30783.70	$3d^{10}4s$	1/2	0.00	324.75	324.8
	$3d^{10}4p$	1/2	30535.32	$3d^{10}4s$	1/2	0.00	327.40	327.4
**Pb**	$6s^26p7s$	1	35287.22	$6s^26p^2$	1	7819.26	363.96	364.0
	$6s^26p7s$	0	34959.91	$6s^26p^2$	1	7819.26	368.35	368.4
	$6s^26p7s$	1	35287.22	$6s^26p^2$	2	10650.33	405.78	405.9

3. Video demonstration of the insensitivity of the obtained spectral lines to water waves

Here we show videos of our experiment in the case when artificial waves are generated in the water cuvette, by a water-immersed mechanical shaker running at two different frequencies. It can be clearly seen from these videos that the extended

light string in this approach overcomes the limitation imposed by the linear diffraction of the laser beam in the conventional nanosecond laser induced breakdown spectroscopy.

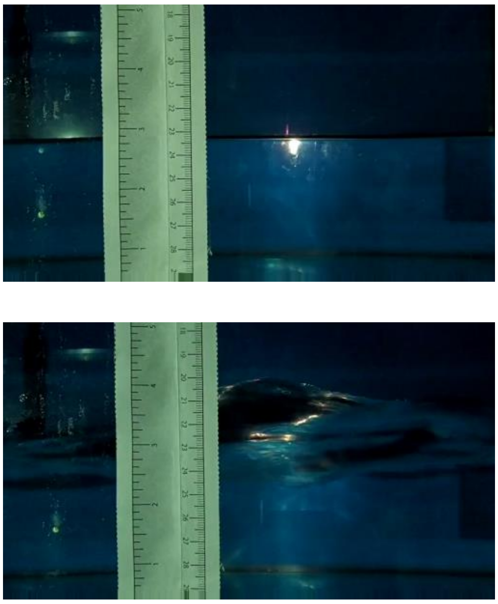

Vibrational Frequency: f = 43 min^{-1}

Vibrational Frequency: f = 120 min^{-1}

Supplement Video S1 (opens by double click). Videos of our experiment with undulating water surface. Artificial water waves are generated by a water-immersed mechanical shaker running at the vibrational frequency f = 43 min^{-1} (top) and f =120 min^{-1} (bottom).

4. Scalability of our approach to the realistic standoff distances

In the long-range implementation of the measurement of water pollutants, due to the phenomenon of intensity clamping in the laser filament, the level of the optical signal at the point of emission on the water surface will be similar to that in our laboratory-scale demonstration. To maintain the same level of the optical signal

detected from a distance, the diameter of the signal-collection aperture needs to be scaled approximately in proportion to the distance. In our experiments, we detect the emission from a 1.1 meter distance, using a 5.08 centimeter-diameter collection lens. Collection of the same order-of-magnitude signal from a 100 meter distance would require a meter-scale collection optic, which is within the feasibility range. Generation of multiple filaments would help improve the signal level. Further enhancements of the detection sensitivity could be achieved through the application of dual laser-pulse and dual-wavelength excitation, gating optimization and the optimization of spectrometer resolution for the detection of particular spectral signatures of interest.
